# Supplementary material for: Effect of Upper Airway Stimulation in Patients with Obstructive Sleep Apnea (EFFECT): A Randomized Controlled Crossover Trial
Source: J Clin Med. 2021 Jun 29;10(13):2880. doi: 10.3390/jcm10132880 (PMC8269272; doi:10.3390/jcm10132880)
Supplement: Supplementary file 1 [file jcm-10-02880-s001.zip › jcm-1229739-supplementary.pdf]

Journal of
Clinical Medicine

MDPI

Supplementary Materials: The following are available online at https://www.mdpi.com/article/10.3390/jcm10132880/s1, **Figure S1**: Study flow of participants from baseline through completion after the 2-week visit, **Table S1**: Sleep Architectural Differences in Response to Upper Airway Stimulation versus Sham. **Table S2**: Sleep Architectural Differences in Response to Upper Airway Stimulation versus Sham

Figure S1: Study flow of participants from baseline through completion after the 2-week visit.

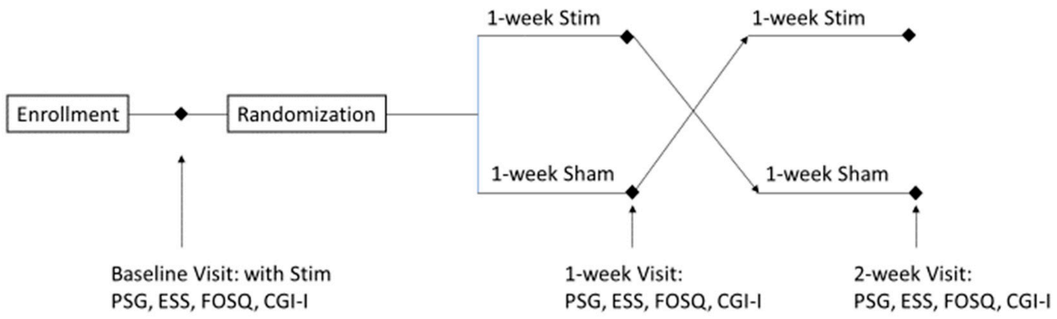

Table S1: Sleep Architectural Differences in Response to Upper Airway Stimulation versus Sham. Mean (95% CI)

Parameter	Stim	Sham	Difference	p-value
AHI (events per hour)	0.6 (-1.8, 2.9)	16.1 (13.7, 18.4)	-15.5 (-18.3, -12.8)	<.001
ODI (events per hour)	0.6 (-1.9, 3.0)	12.7 (10.3, 15.2)	-12.2 (-14.8, -9.6)	<.001
Total time in bed (in hours)	5.8 (-6.5, 18.2)	5.4 (-6.9, 17.7)	0.4 (-12.2, 13.0)	0.948
Total sleep time (in hours)	3.3 (-9.3, 16.0)	11.6 (-1.1, 24.2)	-8.2 (-23.3, 6.9)	0.282
Sleep efficiency (in %)	0.2 (-2.2, 2.5)	2.5 (0.1, 4.9)	-2.3 (-5.4, 0.7)	0.129
N1 total duration (in minutes)	-3.8 (-9.0, 1.4)	2.2 (-3.0, 7.4)	-6.0 (-11.2, -0.8)	0.024
N2 total duration (in minutes)	7.3 (-34.7, 49.3)	42.9 (0.9, 84.9)	-35.6 (-90.9, 19.7)	0.204
N3 total duration of slow wave sleep (in minutes)	2.4 (-6.8, 11.5)	-1.5 (-10.6, 7.7)	3.9 (-7.3, 15.0)	0.494
REM total duration(in minutes)	0.2 (-4.8, 5.3)	0.7 (-4.3, 5.8)	-0.5 (-5.2, 4.2)	0.834
WAKE total duration (in minutes)	-5.3 (-17.5, 6.9)	-5.7 (-17.9, 6.6)	0.4 (-13.9, 14.7)	0.958
Apnoea index (in events per hour)	0.5 (-1.2, 2.3)	8.9 (7.2, 10.7)	-8.4 (-10.6, -6.2)	<.001

Parameter	Stim	Sham	Difference	p-value
AHI in supine position in events per hour)	2.2 (-2.3, 6.6)	23.8 (19.4, 28.2)	-21.6 (-27.2, -16.0)	<.001
Sleep time in supine position (in minutes)	-11.7 (-29.9, 6.6)	-10.3 (-28.6, 7.9)	-1.4 (-22.9, 20.1)	0.898
AHI in non-supine position (in minutes)	-0.1 (-3.2, 2.9)	3.1 (0.1, 6.1)	-3.3 (-6.4, -0.1)	0.044
Sleep time in non-supine position (in minutes)	4.5 (-25.5, 34.5)	1.9 (-28.4, 32.3)	2.6 (-30.6, 35.8)	0.876
AHI in REM sleep (events per hour)	2.0 (-1.6, 5.6)	17.1 (13.5, 20.6)	-15.1 (-19.7, -10.5)	<.001
AHI in non-REM sleep (events per hour)	0.0 (-2.4, 2.5)	15.7 (13.3, 18.2)	-15.7 (-18.5, -12.8)	<.001
Central Apnoea Index (events per hour)	0.1 (-0.1, 0.4)	0.3 (0.0, 0.5)	-0.1 (-0.4, 0.1)	0.285
Mixed Apnoea Index (events per hour)	0.1 (-0.3, 0.4)	0.3 (-0.1, 0.6)	-0.2 (-0.6, 0.2)	0.355
Central Mixed Apnoea Index (events per hour)	-0.0 (-0.8, 0.7)	0.4 (-0.3, 1.1)	-0.4 (-1.2, 0.4)	0.283
Hypopnoea Index (events per hour)	0.0 (-1.6, 1.6)	7.0 (5.4, 8.6)	-7.0 (-8.9, -5.1)	<.001
Percentage of Central/Mixed of total AHI (in %)	0.4 (-2.0, 2.9)	0.5 (-1.9, 3.0)	-0.1 (-2.7, 2.6)	0.955
Minimal measured SaO_2 (in %)	-0.9 (-1.9, 0.2)	-4.0 (-5.0, -3.0)	3.1 (2.1, 4.2)	<.001
Mean SaO_2 (in %)	-0.2 (-0.9, 0.4)	-0.5 (-1.2, 0.1)	0.3 (-0.5, 1.1)	0.493
Total time SaO_2 <90%	2.4 (-1.7, 6.4)	9.0 (4.9, 13.0)	-6.6 (-11.2, -2.0)	0.005
Percentage of TIB SaO_2 <90%	0.3 (-1.0, 1.5)	2.3 (1.0, 3.5)	-2.0 (-3.3, -0.7)	0.004
Arousal Index (events per hour)	1.9 (-1.1, 4.8)	2.2 (-0.7, 5.2)	-0.3 (-4.2, 3.5)	0.861

Table S2: The Clinical Global Impression Changes in Response to Upper Airway Stimulation versus Sham

CGI by Treatment Group	% (n/N)
**Stim**	
1 - Very much improved	39.5% (34/86)
2 - Much improved	27.9% (24/86)
3 - Minimally improved	9.3% (8/86)
4 - No change	19.8% (17/86)
5 - Minimally worse	3.5% (3/86)
**Sham**	
1 - Minimally improved	2.3% (2/87)
2 - No change	6.9% (6/87)
3 - Minimally worse	19.5% (17/87)
4 - Much worse	37.9% (33/87)
5 - Very much worse	33.3% (29/87)

CGI=clinical global impression.
